# Ultrasound-Assisted Deep Eutectic Solvent Extraction of Polysaccharides from Anji White Tea: Characterization and Comparison with the Conventional Method

**DOI:** 10.3390/foods12030588

**Published:** 2023-01-30

**Authors:** Bing Xia, Qi Liu, Da Sun, Yang Wang, Wenjun Wang, Donghong Liu

**Affiliations:** 1 National-Local Joint Engineering Laboratory of Intelligent Food Technology and Equipment, Zhejiang Key Laboratory for Agro-Food Processing, Zhejiang Engineering Laboratory of Food Technology and Equipment, Fuli Institute of Food Science, College of Biosystems Engineering and Food Science, Zhejiang University, Hangzhou 310058, China; 2 Zhejiang Tea Group Co., Ltd., Hangzhou 310003, China; 3 Zhejiang Institute of Economics and Trade, Hangzhou 310018, China; 4 Innovation Center of Yangtze River Delta, Zhejiang University, Jiashan 314100, China; 5 Ningbo Research Institute, Zhejiang University, Ningbo 315100, China

**Keywords:** Anji white tea, ultrasound-assisted deep eutectic solvents, polysaccharide, physicochemical properties, biological properties

## Abstract

Deep eutectic solvent as a new green and safe solvent system has attracted more and more attention in recent years. In this study, three deep eutectic solvents (DES) were combined with ultrasound irradiation to extract tea polysaccharides (TPs) from Anji white tea, which was compared with conventional hot water extraction (HW). The physicochemical, structural, and biological properties of TPs extracted by ultrasound-assisted DES and hot water (HWP) were further investigated. Results showed that the DES system composed of choline chloride and 1,6-hexanediol (CH) with the molar ratio of 1:2 exhibited the optimal extraction yield (19.18%) and in vitro antioxidant activities for TPs (CHP). Furthermore, compared to the HWP, the CHP had a higher extraction yield and total carbohydrate content and a lower molecular weight. Monosaccharide composition analysis displayed that the molecular structure of CHP exhibited more arabinose but less glucose, mannose, galacturonic acid, and glucuronic acid than HWP. Little difference was observed in the preliminary structural characteristics between HWP and CHP from Fourier transform infrared analysis. Besides, CHP possessed better α-glucosidase inhibitory and hypoglycemic activity in L6 cells than HWP. Therefore, the ultrasound-assisted DES extraction method can be a promising strategy for extracting TPs with excellent bioactivities for future applications in functional foods.

## 1. Introduction

Tea (*Camellia sinensis* (L.) O. Kuntze) is a non-alcoholic beverage with a long history worldwide. As the origin of tea, China is the largest tea producer in the world [1]. Generally, Chinese tea can be divided into six groups based on processing methods and sensory qualities: Green tea, black tea, white tea, yellow tea, dark tea, and oolong tea [2]. “Anji Baicha” or called Anji white tea, produced in Anji County, Zhejiang Province, China, belongs to the green tea category and is not fermented. It is an albino mutant, which is common in the genetics of higher plants [3].

Tea polysaccharides (TPs) are general terms for active polysaccharides extracted from tea leaves. They could contain acidic and neutral polysaccharides, and their molecular weights are typically more than 40,000 Da. TPs are critical bioactive components in tea, with a lot of biological activities, such as hypoglycemic effect, anti-tumor, antioxidant, anti-inflammatory, etc. [4,5].

Many studies have proved that the properties of TPs were affected by many factors, such as variety, origin, growth site of leaves, and age of tea plants [6]. Even with the same raw materials, TPs’ extraction rate, physical and chemical properties, and biological activities would differ if different separation and extraction methods were adopted [7,8]. As a traditional extraction method, hot water extraction (HW) usually takes the disadvantages of low efficiency, long time, and high temperature [9], while a lot of studies have found that microwave and ultrasound-assisted technology can maintain the biological activity of TPs and improve the yield [10]. Deep eutectic solvent (DES) has recently attracted much attention as a new green and safe solvent system that is readily biodegradable [11,12]. This solvent system could be adjusted to a broad polarity range, dissolving different targeted compounds. After combining with ultrasonic technology, a high extraction rate could be achieved, which was reported for the polysaccharides extraction from *Indocalamus tessellatus* leaves and *Radix Bupleuri* [13,14].

In this study, “Baiye No.1”, a typical Anji white tea, was used as the object to extract TPs by ultrasound-assisted DES extraction. This study aimed to (1) optimize the extraction process for a higher yield, (2) characterize the physicochemical, structural, and biological properties of TPs, (3) compare ultrasound-assisted DES extraction with conventional HW. This study contributes to developing an efficient and environmentally friendly method of extracting polysaccharides from Anji white tea, which has potentially better bioactivity for future research in functional foods.

## 2. Materials and Methods

### 2.1. Sample Pretreatment

“Baiye No.1” Anji white tea (“Anji Baicha”) was collected in April 2021 from Anji County, Zhejiang Province, China. The sampling site is situated between 119°14′–119°53′ E and 30°23′–30°53′ N. The leaves were washed, sun-dried, and kept in aluminum foil bags at room temperature. The dried white tea leaves were mechanically ground and passed through 50 mesh sieves (<0.3 mm). Before the extraction, they were soaked with 80% ethanol for 2 h to remove impurities such as pigments and dried in the fume hood with ventilation.

### 2.2. Preparation of DES

Three types of DES, including choline chloride-ethylene glycol (CE), choline chloride-1,6-hexanediol (CH), and choline chloride-1,4-butanediol (CB), were made by the molar ratio of 1:2, 1:2, and 1:4 for the components, respectively. Pure water with a specific weight portion (10–30%, *w*/*w*) for each final DES (Table 1) was added to the mixture and heated with a stirring bar in a water bath at 50 °C in a bottle until a uniform and clear solution was formed.

### 2.3. Extraction of TPs

#### 2.3.1. Hot Water Extraction

High temperatures can accelerate the dissolution of polysaccharides from the cell wall and make them more soluble in water. The temperature of HW was 75 °C, and the extraction time was 4 h with a stirring rate of 300 rpm. The extracting solution was mixed with four times the volume of 95% ethanol to precipitate TPs. The precipitate was filtered, and then the precipitate was freeze-dried. Then, the solid was obtained after centrifuging at 9000× *g* (20 min, 4 °C) and dried at 40 °C. Finally, the solid was redissolved in deionized water and dialyzed at 4 °C for 48 h. Thereafter, the TPs solution was lyophilized to obtain the purified TPs (HWP), which was sealed in valve bags and stored at 20 ℃.

#### 2.3.2. DES-Based Ultrasound-Assisted Extraction

Five grams of sample was mixed with 150 mL of DES solution. The extraction process was conducted in an ultrasonic bath (35 kHz, 50 W) at 25 °C, following the extraction conditions in Table 1. After the extraction, the mixture was centrifuged (9000× *g* for 20 min under 4 °C), and the filtrate was coagulated using four times the volume of 95% ethanol. The purified TPs would be obtained using the same method as HW, and expressed as CEP, CHP, CBP extracted from three DES types (CE, CH, CB), respectively [15].

### 2.4. Experimental Design

A completely randomized factorial design (designed as Table 1) was used to investigate the effects of DES type (CE, CH, CB) [15,16], water content of DES solution (10, 20, 30%) [17,18], and extraction time (20, 40, 60 min) [19] on the yield of TPs. The optimal extraction condition for each DES solvent was compared with HW by analyzing the antioxidant activity of extracted TPs. SAS 9.4 (SAS Inst. Inc., Cary, NC, USA) was used to analyze the data. Analysis of variance (ANOVA) was used to find out the significant effects of individual factors and their interactions, and the least significant difference test was used to test the statistical significance between levels (*p* < 0.05). Furthermore, TPs with the highest yield and antioxidant activity using DES solution were further compared with HWP by physicochemical, structural, and biological properties. Each experiment was performed in triplicate.

### 2.5. Antioxidant Activity of TPs

Four well-recognized methods were used to evaluate the antioxidant activity of TPs, which are composed of 2,2′-azino-bis-(3-ethylbenzothiazoline-6-sulfonic acid) diammonium salt (ABTS) radical scavenging capacity, 1,1-diphenyl-2-picrylhydrazyl (DPPH) radical scavenging capacity, ferric reducing antioxidant power (FRAP), and oxygen radical absorbance capacity (ORAC). 

#### 2.5.1. ABTS Radical Scavenging Activity

The ABTS solution from the ABTS kit (S0119, Beyotime, Nantong, China) was mixed with the oxidant master mix in equal proportions and stored for 12 h before use, and 400 μL of the master mix was mixed with 13.6 mL of 0.01 M phosphate buffer (pH = 7.3, C0221A, Beyotime, Nantong, China) as the working master mix. Ten microliters of the sample solution containing different samples at a concentration of 0.5 mg/mL was added to 200 μL of working master batch and reacted for 5 min to measure at 734 nm using an ultraviolet-visible spectrophotometer (Infinite E Plex, TECAN, Männedorf, Switzerland). Trolox ((±)-6-Hydroxy-2,5,7,8-tetramethylchromane-2-carboxylic acid) was used to compare the ABTS radical scavenging activity with the TPs, and the results would be shown as mg Trolox equivalents (TE)/g TPs [20].

#### 2.5.2. DPPH Radical Scavenging Activity

We added 0.2 mL of TPs water solution (1 mg/mL) to 2.8 mL of DPPH (0.1 M), dissolved in 95% ethanol. The reaction mixture was under vortex oscillation and left under shaded conditions at 20 °C for 30 min to measure at 517 nm using the same ultraviolet-visible spectrophotometer above. The results were reported as mg ascorbic acid equivalents (AAE)/g TPs [21].

#### 2.5.3. FRAP

FRAP reagent was prepared right away, including 100 mL of sodium acetate buffer (300 mM, pH 3.6), 10 mL of 2,4,6-tripyridyltriazine (TPTZ) solution (10 in 40 mM HCl), and 10 mL of FeCl_3_-6H_2_O solution (20 mM). Different TPs solutions (3 mg/mL, 0.1 mL) were mixed with 4.9 mL of FRAP reagent. We measured the resulting mixtures at 593 nm using the same instrument as ABTS and DPPH 10 min later [22]. The results were shown as mg Trolox equivalents (TE)/g TPs.

#### 2.5.4. ORAC

The fluorescence excitation and emission wavelengths were 485 and 538 nm, respectively, using EnSpire software version 4.13 [23]. We added 25 μL of 504 nM fluorescein disodium salt configured in phosphate buffer (pH = 7.3, C0221A, Beyotime, Nantong, China) to an equal volume of 0.2 mg/mL TP sample and incubated in the microplate at 37 °C for 5 min. Each well was then quickly treated with 2,2′-Azobis (2-methylpropionamidine) dihydrochloride (AAPH) solution with PBS buffer (150 μL, 17.07 mM, the final concentration). Fluorescence was recorded in a plate reader with automatic shaking every 2 min for a total of 120 min. The inhibitory capacity was quantified by combining the fluorescence decay data with the area under the integration curve. ORAC were reported as the same unit as ABTS.

### 2.6. Molecular Weight and Its Distribution

The analysis system for determining molecular weight and its distribution consisted of a high-performance liquid chromatograph (Waters 2695, Milford, MA, USA), a guard column (OHpak SB-G, Shodex, Yokohama, Japan), size exclusion columns (SB-806 HQ and SB-804 HQ column, 7.8 × 300 mm, Shodex, Yokohama, Japan), a multiangle laser light scatterer (DAWN HELEOS Ⅱ, Wyatt Technology, Goleta, CA, USA), and a refractive index detector (RID-20A, Shimadzu, Kyoto, Japan), which was called SEC-MALLS-RI system [24]. A 0.15 mol/L NaCl solution (containing 0.02% NaN_3_) was prepared with ultrapure water as the mobile phase, passed through a 0.22 μm aqueous membrane, degassed by ultrasonication for 30 min, and set aside. Three-milligram samples were dissolved in 1 mL mobile phase and filtered by 0.22 μm hydrophilic membrane before the test. The injection volume and column temperature were 50 μL and 25 °C, respectively. The data would be collected using ASTRA 7.1.2 software for 60 min of each sample with the refractive index increment (dn/dc) of 0.138 mL/g.

### 2.7. Monosaccharide Composition Determination

The monosaccharide compositions of TPs were analyzed by acid hydrolysis combined with ion chromatography, according to Zhang et al. reported [23]. Briefly, 2 mg samples were hydrolyzed using 1 mL of 4 mol/L trifluoroacetic acid (TFA) at 110 °C for 8 h. The TFA was eliminated in a nitrogen atmosphere, and the hydrolysis products were filtered through a ɸ 13 mm × 0.22 μm polyethersulfone syringe-driven filter prior to the test. Fucose (Fuc), rhamnose (Rha), arabinose (Ara), galactose (Gal), glucose (Glc), mannose (Man), xylose (Xyl), fructose (fru), galacturonic acid (GalA), and glucuronic acid (GlcA) were used as the monosaccharide standards. The Dionex system (ICS-5000, ThermoFisher, Waltham, MA, USA) with a pulsed electrochemical detector and Carbopac™ PA10 analytical columns at 30 °C was used to determine the monosaccharide contents of prepared hydrolysates and monosaccharide standards. The separation method was performed by isocratic elution with 18 mmol/L sodium hydroxide (NaOH) for 15 min followed by 18 mmol/L NaOH in 100 mM sodium acetate (NaOAc) for the subsequent 35 min.

### 2.8. Fourier Transform Infrared (FT-IR) Spectroscopy

The FT-IR spectrometer (Perkin Elmer Spectrum BX FT-IR) was used to measure the FT-IR spectra of TPs [25]. The ground samples were added to spectral-grade potassium bromide (KBR) powder and were then pressed into 1 mm discs. The results were recorded in the wave number range of 4000–400 cm^−1^.

### 2.9. α-Amylase and α-Glucosidase Inhibition Assays

The inhibition assay of α-amylase was adapted from Chen et al. [26] with some modifications. Briefly, the sample solutions were prepared by phosphate buffer solution (0.1 M, pH 6.9). After incubation at 37 °C for 10 min, 500 μL of 1% soluble starch solution was added to the mixture and incubated for 15 min at 37 °C. Then, 1 mL of 3,5-dinitrosalicylic acid (DNS) solution was added and boiled for 10 min, followed by the addition of 7.5 mL of distilled water to the mixture. The absorbance value was measured at 540 nm, and the inhibition rate was calculated. The inhibition rate of TPs was calculated as follows:

α-Amylase inhibition activity (%) = (A_control_ − A_sample_)/A_control_ × 100%

The inhibitory assay of α-glucosidase was performed according to Karim et al. [27]. First, 20 μL α-glucosidase (1 mg/mL dissolving in 0.05 M PBS, pH 6.9), 20 μL of sample solution (0, 0.0625, 0.0125, 0.25, 0.5, 1, 2 mg/mL), and 50 μL of PBS (0.05 M, pH 6.9) were added to a 96-well plate and placed in a shaking incubator at 37 °C and 500 rpm for 10 min. Then p-nitrophenyl-α-D-glucopyranoside (pNPG) solution (0.05 M, 20 μL) was added and incubated for 20 min. The reaction was terminated by adding 20 μL Na_2_CO_3_ solution (0.2 M). The absorbance of the solution was measured at 405 nm using an enzyme marker.

### 2.10. Glucose Uptake

The glucose uptake assay was adapted from our previous studies [28]. The fully differentiated cells were treated with different concentrations of TPs (0.125, 0.25, and 0.5 mg/mL). The glucose uptake was calculated from the changes in glucose concentrations before and after sample treatment.

### 2.11. Statistical Analysis

All data were expressed as mean ± standard deviation of triplicate measurements. Differences were assessed by one-way analysis of variance with Duncan’s multiple range test.

## 3. Results and Discussion

### 3.1. Optimization of TPs Extraction Process

**Figure 1:**
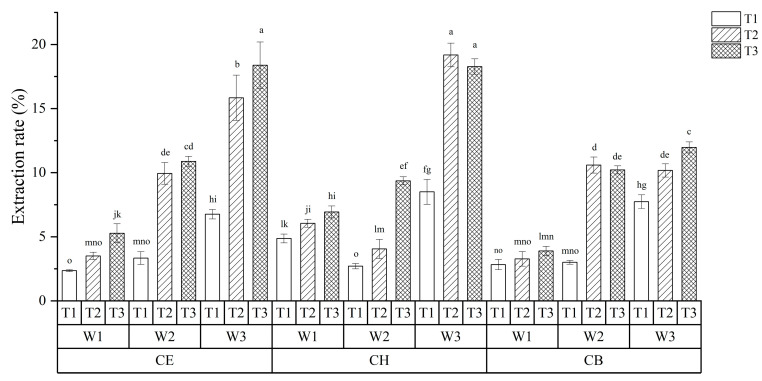
The yield of tea polysaccharides (TPs) under different extraction solvents, ultrasonic time and water content. T: Extraction time, 1, 2, and 3 mean 20, 40, and 60 min. W: Water content, 1, 2, and 3 mean 10%, 20%, and 30%. CE: Choline chloride and ethylene glycol, 1:2 molar ratio; CH: Choline chloride and 1,6 hexanediol, 1:2 molar ratio; CB: Choline chloride and 1,4-butanediol, 1:4 molar ratio; Means with different letters indicate a significant difference (*p* < 0.05).

A completely randomized factorial design was used to investigate the extraction yield of TPs with three DES at different ultrasound extraction times and water contents, and the results are shown in Figure 1. The yield of TPs reached the highest when the water content was 30% for the three low eutectic solvents. When CE was selected as the extraction solvent, the extraction rate of TPs was the highest (18.38%) after 60 min of ultrasonic treatment with a water content of 30%. With respect to CH, the yield of TPs was the highest (19.18%) after 40 min of ultrasound-assisted extraction. Regarding CB, the highest extraction rate of TPs was 11.97%.

The independent and interactive effects of three different factors were studied, and the results are shown in Table 2. There were significant independent and interactive effects among the three factors, which indicated that all three factors, including solvent type, water content of solvent, and ultrasonic time, could significantly affect the extraction yield. What is noteworthy is that the interactive effect of the three factors showed extreme significance, indicating that we could compare all 27 groups together through one-way ANOVA. In this case, according to the results in Figure 1, the three combinations of CE-W3-T3 (CE/30% water content/60 min), CH-W3-T2 (CH/30% water content/40 min), and CH-W3-T3 (CH/30% water content/60 min) had the highest TPs yields, and there was no significant difference among them. Considering energy saving, the optimal extraction process of LPs was CH-W3-T2, including using the solvent of CH, the ultrasonic treatment time of 40 min, and a water content of 30%. The optimized ultrasound-assisted DES method can effectively improve the extraction efficiency of polysaccharides from Anji white tea, which can be chosen as the optimal ultrasound-assisted DES extraction condition for the following research.

### 3.2. The Antioxidant Activity In Vitro

**Figure 2:**
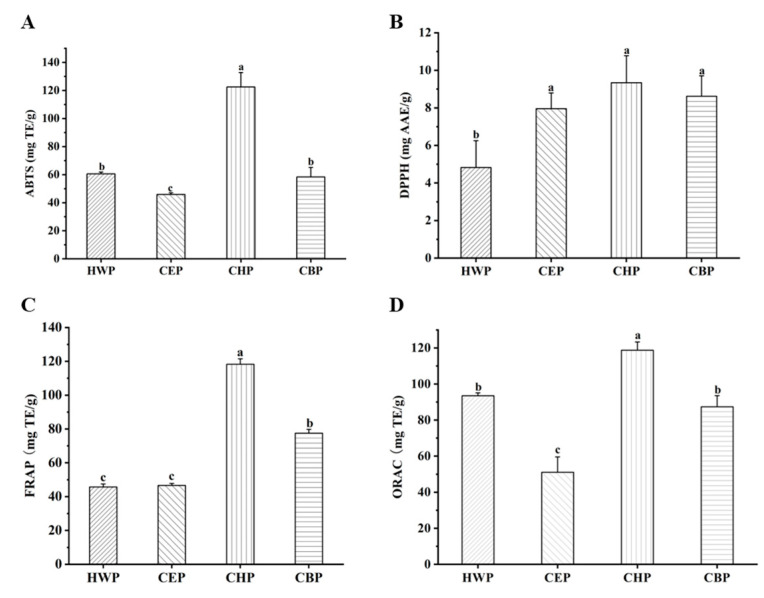
Antioxidant activity determined by ABTS (**A**), DPPH (**B**), FRAP (**C**), and ORAC (**D**) assays of TPs after extraction by different methods. Statistical significance (*p* < 0.05) was indicated with different lowercase letters within each subfigure.

The proper cellular concentrations of reactive oxygen species (ROS) in the human body can function as “redox messengers” in intracellular signaling and regulation. At excess physiological levels, ROS can induce oxidative stress and oxidative damage, thus causing various organ diseases, including cancer, diabetes, and inflammatory diseases [29]. Numerous studies show that antioxidants could reduce oxidative stress and oxidative damage and have a significant effect on preventing and treating diseases. Studies have shown that polysaccharides could scavenge free radicals, thus improving oxidative damage [30]. A series of in vitro activity tests (ABTS, DPPH, FRAP, and ORAC) were carried out to study the antioxidant activities of TPs extracted by different methods (Figure 2). As can be seen from Figure 2A, CHP showed superior activity in ABTS radical scavenging capacity (*p* < 0.05), followed by HWP. As can be seen from Figure 2B, CHP had a slightly higher antioxidant capacity against DPPH radicals, although there was no significant difference compared to other DES-extracted TPs. Moreover, the iron reduction capacity of CHP was better than (*p* < 0.05) that of TPs extracted by other methods (Figure 2C). Interestingly, the ORAC of TPs extracted by different methods showed a similar trend with ABTS and FRAP results (Figure 2D). It is worth noting that the molecular weight of TPs might play an important role in its antioxidant activity. A study performed by Zhu et al. found that weight-average molecular weight (*M_w_*) of TPs from dark tea has a positive effect on their antioxidant activity [31]. In another study, Kang et al. confirmed that a low *M_w_* (42.8 kDa) of maca polysaccharides showed better antioxidant activity than that of a high *M_w_* (93.5 kDa) [32]. Meanwhile, lower *M_w_* of CHP in our trial also had higher antioxidant capacity, indicating the *M_w_* has a significant effect on the antioxidant activity the TPs. It is interesting to note that the viscosity of low-*M_w_* polysaccharides was lower than that of high-*M_w_* polysaccharides, thus making it easier to scavenge free radicals because lower viscosity could help polysaccharides more easily contact with free radicals [31]. These results induced that TPs from Anji white had the ability to relieve oxidative stress via scavenging free radicals, enhancing iron reduction capacity and oxygen radical absorbance capacity. Moreover, CHP possessed the highest antioxidant activities among all tested TPs extracted by ultrasound-assisted DES methods, suggesting that the extraction process indeed affected the antioxidant activity of TPs.

### 3.3. Comparison of HWP and CHP

#### 3.3.1. Extraction Yields and Total Carbohydrate

The extraction yields, total carbohydrate, and monosaccharide composition of HWP and CHP from the Anji white tea are shown in Table 3. The extraction methods remarkably influenced the extraction yields and total carbohydrate content. According to the extraction methods, the extraction yields of CHP (19.18 ± 0.92%) increased by 248.73% compared to that of HWP (5.50 ± 0.62%). In general, the lower extraction yield of HWP obtained by hot water extraction was because the water merely dissolved long-chain soluble polysaccharides [33,34]. However, the ultrasound-assisted extraction appeared to have a high extraction yield owing to the high shear, microjet, and turbulence generated by the rupture of ultrasonic cavitation bubbles to accelerate the extraction efficiency of polysaccharides [35]. Moreover, it should be noted that the components of the DES system could supply or receive external electrons or protons for the formation of hydrogen bonds that induce the dissolution of a wide variety of substances, including polysaccharides, thereby improving the extraction efficiency of polysaccharides [15]. Therefore, ultrasound-assisted DES extraction of polysaccharides had higher extraction efficiency than traditional HW. This finding was in accord with those of Zhang et al. [15], which displayed a lower yield with hot water extraction (10.51%) than with ultrasound-assisted DES extraction (15.98%). In addition, the higher total carbohydrate content was obtained from CHP (72.67 ± 4.99%) via ultrasound assisted DES extraction, and the lower one was obtained from HWP (65.16 ± 6.06%) via HW. It may be attributed to the polysaccharide glycosidic bonds, and the branched chain structure was easily destroyed by the combination of ultrasound and DES [36]. Similarly, Pan et al. [37] found that the total carbohydrate of polysaccharide from *Morchella importuna* extracted by ultrasound-assisted DES (85.27%) was markedly higher than that of HW (57.89%). Collectively, the differences in the extraction yield and the total carbohydrate content may be correlated with the type of extraction method chosen, and ultrasound-assisted DES extraction could be a promising extraction strategy with high extraction yield and total carbohydrate content.

#### 3.3.2. Monosaccharide Compositions

Ion chromatography followed by acid hydrolyzing was used to measure the monosaccharide compositions of TPs, and the results are summarized in Table 3. Depending on the monosaccharide standards, both two TPs possessed ten types of monosaccharides, which were composed of Rha, Ara, Fuc, Gal, Glc, Man, Xyl, Fru, GalA, and GlcA. These findings demonstrated that TPs were acidic heteropolysaccharides. By contrast, Table 3 illustrated that the monosaccharide contents of the HWP and CHP differed in variation. The major monosaccharides in HWP and CHP were Ara (19.48–28.29%), Gal (26.39–27.27%), Glc (7.30–8.52%), Fru (9.74–10.94%), GalA (10.01–12.21%), and GlcA (7.44–13.77%). It was evident that compared with the hot water extraction, the ultrasound-assisted DES extraction could significantly decline the content of GalA and GlcA, indicating a decrease in uronic acid content in CHP. Interestingly, the content of Ara and Gal in CHP was higher than that of HWP, indicating that CHP may possess large amounts of arabinogalactan, which were extensively distributed in the cell walls of plants. These results may be due to the fact that the ultrasound-assisted DES extraction could lead to hydrolytic disruption of polysaccharide chains and breakage of intermolecular hydrogen bonds, which affects the composition of monosaccharides [38]. Similarly, Zhang et al. reported that three crude polysaccharides extracted from *Indocalamus tessellatus* leaves by hot water, ultrasonic and ultrasonic-assisted DES exhibited the same monosaccharide components but different molar ratios [13]. Thus, the different extraction methods had different impacts on the molar ratio of the monosaccharide composition of TPs.

#### 3.3.3. Molecular Weight

The size-exclusion chromatography combined with the multiangle laser light scattering and the refractive index detector (SEC-MALLS-RI) analysis was used to investigate *M_w_*, number-average molecular weight (*M_n_*), and molecular-weight distribution (*M_w_*/*M_n_*) of HWP and CHP. The procured findings are illustrated in Table 4. Overall, two fractions were observed in CHP and HWP. More specifically, HWP existed two peaks with the *M_w_*s of 2.558 × 10^6^ Da (fraction 1), and 3.020 × 10^5^ Da (fraction 2), and the higher *M_w_* fraction of ~66.80% was the dominant based on the relative peak area. Furthermore, CHP showed two peaks with the *M_w_*s of 9.016 × 10^5^ Da (fraction 1) and 5.837 × 10^4^ Da (fraction 2), and the lower *M_w_* fraction of ∼93.30% was the main composition. Alternatively, the *M_w_*s of CHP were significantly lower than that of HWP. Interestingly, the lower *M_w_* values of bioactive polysaccharides may present higher antioxidant capacities [39]. In the current work, the differences in *M_w_* of polysaccharides were correlated with different extraction techniques. The cavitation effects generated by ultrasound treatment broke the glycosidic chain of high-*M_w_* polysaccharides, thus reducing their *M_w_* [40]. Similarly, Wang et al. demonstrated that the *M_w_* of yellow tea polysaccharides precipitated by 30% ethanol decreased from 37.70 to 15.10 kDa after ultrasonic treatment [41]. Moreover, Wu et al. [42] found that the *M_w_* of lotus leaves polysaccharides obtained by DES extraction (4.03 × 10^4^ Da) was extremely lower than that of hot water extraction (12.90 × 10^4^ Da, 4.70 × 10^4^ Da, and 3.43 × 10^4^ Da). Thus, ultrasound and DES treatment led to the transformation of long-chain polysaccharide molecules into smaller molecules. As displayed in Table 4, *M_n_* exhibited a consistent trend with the *M_w_* values. In addition, *M_w_*/*M_n_* of TPs indicated that the ultrasound-assisted DES extraction of CHP had relatively higher homogeneity than that of HWP extracted with hot water.

#### 3.3.4. FT-IR Spectra

**Figure 3:**
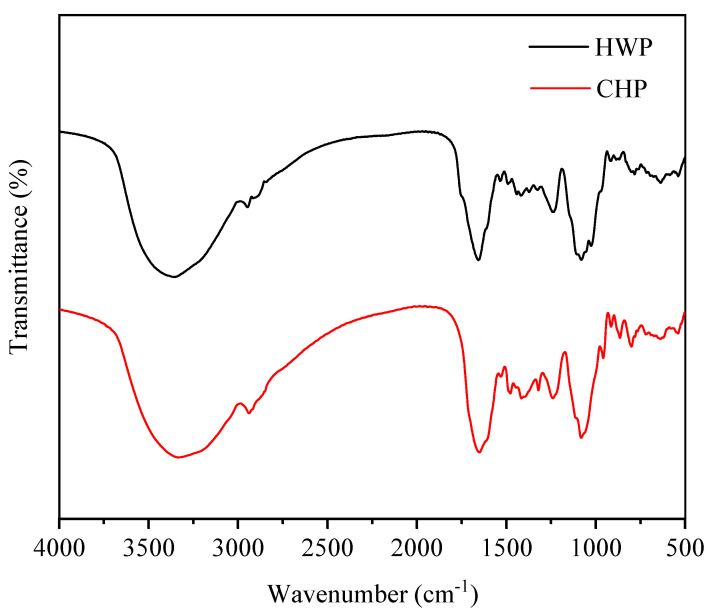
FT-IR spectra of HWP and CHP.

The preliminary structural information and the main functional groups of polysaccharides were investigated by FT-IR spectroscopy. Figure 3 displays that all samples exhibited similar IR spectra, indicating they possessed similar preliminary chemical structure characteristics. Precisely, the absorption peak at around 3435 and 2947 cm^−1^ were the two typical characteristic peaks of polysaccharides, which were triggered by the stretching vibrations of O-H and C-H groups, respectively [43]. The absorption peaks near 1650 and 1448 cm^−1^ were related to the stretching vibrations of asymmetric and symmetric C=O groups, respectively [44], further confirming that TPs was an acidic polysaccharide. Furthermore, the absorption peak at approximately 1249 cm^−1^ was owing to the C-O-C groups stretching vibration. The absorption peak near 1064 cm^−1^ was correlated with the presence of a pyranose ring [45]. Collectively, the characteristic functional groups of TPs were not affected by the extraction methods. Similar findings were reported by Chen et al. [46] and Yan et al. [33].

#### 3.3.5. The α-Amylase and α-Glucosidase Inhibitory Effect

**Figure 4:**
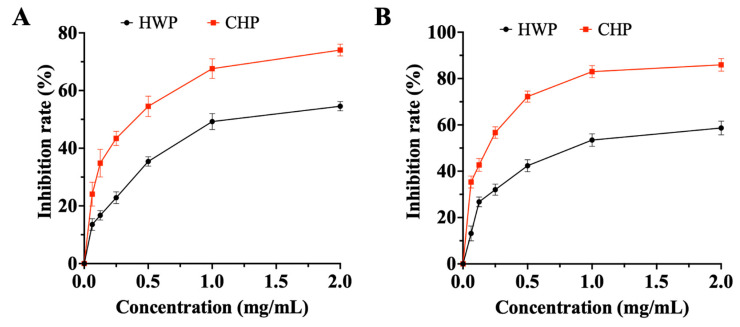
The inhibitory effects of TPs on α-amylase (**A**) and α-glucosidase (**B**).

Blood glucose homeostasis is important for the human body to ensure normal biological function. The loss of glucose homeostasis could result in many metabolic syndromes and complications, such as obesity and diabetes mellitus [47]. Acute glucose elevations after meal ingestion are associated with a variety of glucose-mediated tissue defects. The enzymes α-amylase and α-glucosidase are involved in the carbohydrate and oligosaccharides digestion. Thus, inhibition of α-amylase and α-glucosidase activity can prolong the overall digestion time of carbohydrates and oligosaccharides, thereby improving postprandial hyperglycemia [48]. Studies have shown that tea is widely used as a health drink to prevent and treat hyperglycemia [49]. The hypoglycemic effects of TPs have attracted much attention since the incidence of diabetes is increasing worldwide. Therefore, the α-amylase and α-glucosidase inhibitor activity of TPs were further investigated. The results showed that the inhibitory rate of α-amylase of HWP is lower than that of CHP (Figure 4A). The IC_50_ values of HWP and CHP for α-amylase were 1.52 and 0.36 mg/mL, respectively. The maximal inhibition rate of α-amylase reached 74.04% when the concentration of CHP at 2.0 mg/mL. According to the study of Wang et al., the higher α-glucosidase inhibitory activity could attribute to the specific structure of monosaccharide analogs with five- or six-membered carbon rings [50]. In addition, uronic acid usually exists in the form of five- or six-membered carbon rings. As shown in Figure 4B, HWP and CHP had dose-dependent inhibitory on α-glucosidase activities. The IC_50_ value of HWP and CHP for α-glucosidase was 0.91 and 0.18 mg/mL, respectively. Moreover, CHP showed significantly higher inhibitory effects on α-glucosidase with inhibitory rates of 85.92% than that of HWP was 58.65% when their concentration was at 2.0 mg/mL. Previous studies also reported that green TPs extracted by HW exhibited anti-α-glucosidase activity, but the inhibitory rate was about ~25% at the concentration of 2.0 mg/mL [51]. The digestive enzyme inhibitory potency of the tea polysaccharides could be mainly contributed by its contents. Additionally, α-glucosidase inhibitory activity of polysaccharides is also attributed to its special characteristic of the higher content of Ara and loose chain structure [52], which might account for the similarly excellent activity of CHP that was richer in carbohydrate content. The inhibition of TPs on α-amylase and α-glucosidase exhibited differences that may be related to its structure, type of glycoside bond, and binding metal ions [53]. Accumulated evidence has shown that the activities of polysaccharides are closely related to their molecular weights. According to the study of Xu et al. [51], TPs with relatively small molecular weights exhibited better hypoglycemic activity, which was consistent with our findings that the lower *M_w_* of TPs (CHP) possessed better hypoglycemic activity. These results further indicated that Anji white TPs extracted by CH had potential hypoglycemic activity.

#### 3.3.6. Hypoglycemic Effect of TPs in L6 Cells

**Figure 5:**
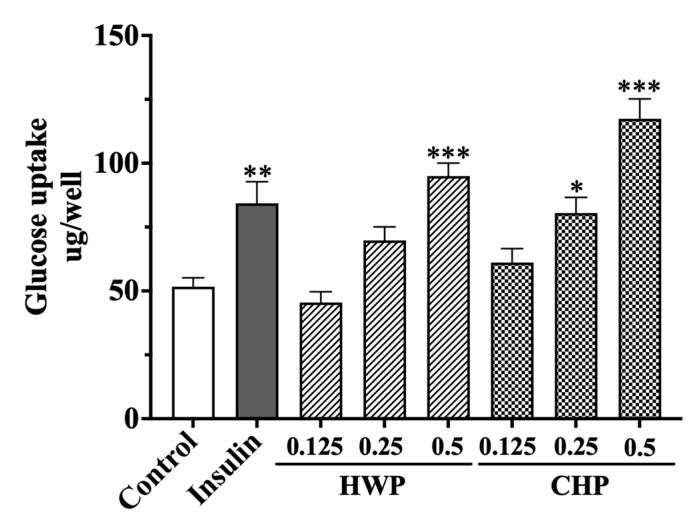
Effects of different concentrations of TPs (0.125, 0.25, and 0.5 mg/mL) extracted by different methods on glucose uptake in L6 cells. Each value represents the mean ± SEM of three independent experiments. * *p* < 0.05, ** *p* < 0.01, and *** *p* < 0.001.

Skeletal muscle is the primary tissue that can ingest and dispose of about 80% of total glucose in the body [54]. L6 cell is widely used to study the glucose uptake activity of different compounds [51,55]. The hypoglycemic activity of HWP and CHP were evaluated in L6 cells. As shown in Figure 5, cells treated with 1 μmol/L insulin resulted in a remarkably increased glucose uptake in L6 cells (1.63-fold, *p* < 0.01; Figure 5) compared with the untreated control group. Meanwhile, cells treated with CHP could also enhance the glucose uptake compared to the control group in a dose-dependent manner (0.125, 0.25, and 0.5 mg/mL). Notably, compared to the control group, TPs (0.5 mg/mL) in HWP and CHP groups elevated the glucose uptake of myotubes with a fold change of 1.84 ± 0.31 (*p* < 0.001) and 2.27 ± 0.18 (*p* < 0.001), respectively. The results were in agreement with the study of Xu, in which green TPs extracted by HW showed better hypoglycemic activity in L6 cells [51]. In addition, previous studies have confirmed that polysaccharides from guava leaves can significantly reduce fasting glucose in diabetic mice [56]. Our results indicated that the polysaccharides in Anji white tea extracted by CH were better than that of the HW method, which confirmed that the extraction process could affect the hypoglycemic activity of TPs. Moreover, these results suggest that the CHP in Anji white tea had the best capability to prevent and manage hyperglycemia-related diseases.

## 4. Conclusions

The present study investigated three deep eutectic solvent systems to extract tea polysaccharides from Anji white tea. The highest extraction yield (19.18%) and antioxidant activities of TPs were obtained with the solvent system of choline chloride and 1, 6-hexanediol at the molar ratio of 1:2 under the solvent water content of 30% and ultrasonic time of 40 min. Compared to the HW (yield, 5.50%), CH was much more efficient for extracting polysaccharides from Anji white tea. Meanwhile, CHP possessed a higher total carbohydrate (72.67%) and a lower *M_w_* (9.016 × 10^5^ Da, 5.837 × 10^4^ Da) than HWP, but both exhibited the structure with the same functional groups and monosaccharide types. The molecular structure of CHP exhibited more Ara while less Glc, Man, GalA, and GlcA than HWP. Additionally, CHP showed excellent α-amylase and α-glucosidase inhibitory activity, and hypoglycemic activity in L6 cells, indicating better potential applications in functional foods. Hence, ultrasound-assisted deep eutectic solvent extraction proved to be a green and effective method for extracting polysaccharides with high bioactivities from Anji white tea.

## Figures and Tables

**Table 1:** Factor design of ultrasonic-assisted deep eutectic solvent extraction. CE: Choline chloride and ethylene glycol, 1:2 molar ratio; CH: Choline chloride and 1,6 hexanediol, 1:2 molar ratio; CB: Choline chloride and 1,4-butanediol, 1:4 molar ratio.

Levels	Solvent	Extraction Time (min)	Water Content (*w*/*w*, %)
1	CE	20	10
2	CH	40	20
3	CB	60	30

**Table 2:** The independence or interactive effects of deep eutectic solvents (sol), water contents (water), and ultrasound extraction times (time) on tea polysaccharides yield were analyzed based on analysis of variance (ANOVA) results.

Group	DF	Sum_sq	MS	F-Value	*p*-Value
sol	2	48.368496	24.18425	48.48	<0.0001
water	2	1052.052652	526.0263	1054.45	<0.0001
time	2	513.006052	256.503	514.17	<0.0001
sol-water	4	164.575719	41.14393	82.48	<0.0001
sol-time	4	24.676096	6.169024	12.37	<0.0001
water-time	4	137.223385	34.30585	68.77	<0.0001
sol-water-time	8	93.045289	11.63066	23.31	<0.0001

DF: Degree of freedom; Sum_sq: Sums of squares; MS: Mean square.

**Table 3:** Extraction yield, total carbohydrate, and monosaccharide composition of HWP and CHP.

Sample	HWP	CHP
Extraction yield (%)	5.50 ± 0.62 ^b^	19.18 ± 0.92 ^a^
Total carbohydrate (wt%)	65.16 ± 6.06 ^b^	72.67 ± 4.99 ^a^
Monosaccharide constituents (molar ratios) (mol%)		
Fuc	0.33 ± 0.03 ^b^	0.45 ± 0.00 ^a^
Rha	2.78 ± 0.65 ^a^	2.88 ± 0.04 ^a^
Ara	19.48 ± 1.62 ^b^	28.29 ± 0.22 ^a^
Gal	26.39 ± 2.25 ^a^	27.27 ± 0.12 ^a^
Glc	8.52 ± 0.94 ^a^	7.30 ± 0.09 ^b^
Man	4.27 ± 0.18 ^a^	3.34 ± 0.15 ^b^
Xyl	2.49 ± 0.26 ^a^	2.20 ± 0.16 ^a^
Fru	9.74 ± 1.18 ^a^	10.94 ± 0.06 ^a^
GalA	12.21 ± 2.55 ^a^	10.01 ± 0.25 ^b^
GlcA	13.77 ± 4.05 ^a^	7.44 ± 0.18 ^b^

Values with different superscript letters in the same row indicate a significant difference (*p* < 0.05).

**Table 4:** Molecular characteristics of HWP and CHP.

Sample	Fraction	*M_w_* (Da)	*M_n_* (Da)	Mass Fraction (%)	*M_w_*/*M_n_*
HWP	1	2.558 × 10^6^ (±0.989%)	9.840 × 10^4^ (±1.380%)	66.80	2.600 (±1.697%)
	2	3.020 × 10^5^ (±2.967%)	2.922 × 10^5^ (±3.156%)	33.20	1.034 (±4.332%)
CHP	1	9.016 × 10^5^ (±3.174%)	6.384 × 10^5^ (±2.997%)	6.70	1.412 (±4.365%)
	2	5.837 × 10^4^ (±4.434%)	5.199 × 10^4^ (±5.037%)	93.30	1.123 (±6.711%)

## Data Availability

The data presented in this study are available on request from the corresponding author.
